# The De-Scent of Sexuality: Should We Smell a Rat?

**DOI:** 10.1007/s10508-019-01591-z

**Published:** 2019-12-05

**Authors:** Jackson Clive, William Wisden, Vincent Savolainen

**Affiliations:** 1 Department of Life Sciences, Silwood Park Campus, Imperial College London, Ascot, SL5 7PY UK; 2 Department of Life Sciences, South Kensington Campus, Imperial College London, London, UK

In their Target Article, Pfau, Jordan, and Breedlove (2019) proposed a connection between the transient receptor potential cation channel 2 gene (*TRPC2*) and same-sex sexual behavior (SSSB) in primates. This novel theory is an attractive prospect for researchers investigating sexuality in the natural world. The proposal relies on evidence from proximate mechanism studies of *TRPC2* knockout (KO) experiments in mice, in which non-functional *TPRC2* alters the development of an olfactory sensory structure called the vomeronasal organ (VNO), resulting in an increase in SSSB in both males and females (Axel et al., 2002; Kimchi, Xu, & Dulac, 2007). In combination with an examination of *TRPC2* sequence data and evolutionary relationships across primates, Pfau et al. proposed some hypotheses for the fitness consequences of SSSB in primates. Pfau et al. speculated that primates with multi-male/multi-female societies may have evolved via improved social cohesion facilitated by an increase in SSSB, mediated by non-functional *TRPC2*, and/or pleiotropy between increased SSSB and reduced same-sex aggression. Here, although we support some of these ideas by providing a more complete examination of *TRPC2* in primates, we also advocate greater caution when interpreting available data on SSSB.

## Multiple Genes Underpin Same-Sex Sexual Behavior

Before discussing the evidence for a potential link between the *TRPC2* gene and SSSB in primates, and indeed all mammals, it is essential to clarify that any such link ought not to be interpreted as promoting a single “gay gene” theory of homosexuality (i.e., same-sex sexual partner preferences) and SSSB. Firstly, there is already a growing body of evidence for an epigenetic and polygenic underpinning of homosexuality and SSSB (Ratnu, Emami, & Bredy, 2017; Rice, Friberg, & Gavrilets, 2016; Sanders et al., 2017). Secondly, since it is the absence of functional *TRPC2* that appears to facilitate heightened SSSB, it seems that the gene is not itself driving SSSB, but instead that it is perhaps underpinning same-sex *aversion*, which is inversely related but not inherently antithetical to SSSB. Finally, it is evident from the presence of SSSB in animals with functioning *TRPC2* and VNO (for example, in rodents, spider monkeys, and bison; see Bagemihl, 1999; Busia, Denice, Aureli, & Schaffner, 2018; Sommer & Vasey, 2006) that the effect of *TRPC2* pseudogenization (i.e., loss of function due to a premature stop codon) cannot completely explain the expression of the behavioral phenotype for SSSB.

Further support for the polygenic nature of SSSB derives from comparisons of *TRPC2* with another gene, tryptophan hydroxylase 2 (*TPH2*). Initial work suggested that *TPH2*, which facilitates 5-HT neurotransmitter synthesis and is critical for serotonergic neuron function, had a strong role in the modulation of SSSB (Liu et al., 2011; Zhang, Liu, & Rao, 2013). These researchers reported that *TPH2* KO males showed no significant preference for either males or females, in addition to showing significant increases in SSSB. This result was contrasted with *TRPC2* KO males that, by comparison, exhibited only a reduced preference for females relative to males (Axel et al., 2002; Liu et al., 2011). However, subsequent attempts to replicate the effects of *TPH2* KO have questioned the connection between functional *TPH2* and sexual partner preference (as a different type of sexual preference behavior experiment showed that both *TPH2* KO and wild-type males preferred females), although SSSB nevertheless increased in *TPH2* KO males (Angoa-Pérez et al., 2015). Importantly, this contention brings into question the assumption of a direct inverse relationship, or degree of non-independence, between opposite-sex sexual behavior and SSSB. Attraction or propensity for SSSB might plausibly be independent of opposite-sex attraction and behavior, whereas a sexual preference for one sex versus the other must inherently be directly and inversely dependent.

## *TRPC2* in the VNO, and Same-Sex Sexual Behavior Across Primates

**Fig. 1:**
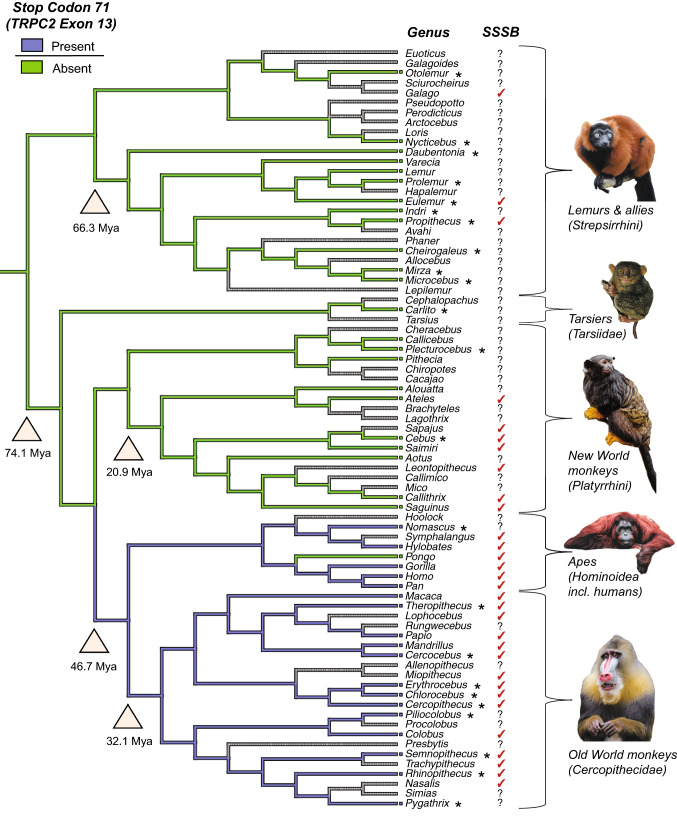
Distribution of a premature stop codon in exon 13 of *TRPC2* and SSSB across the phylogeny of primates. We found that the earliest stop codon in exon 13 of *TRPC2* to appear in primates was at position 71, along the branch leading to Old World monkeys and apes (blue = stop codon present; green = stop codon absent; gray = unknown). The presence of SSSB was determined from behavioral reports, with an uncertain status (indicated by a question mark) applied to genera without confirmed SSSB in the wild (Bagemihl, 1999; Carosi & Visalberghi, 2002; Chandler, 1975; Fang, Dixson, Qi, & Li, 2018; Fox, 2001; Grueter & Stoinski, 2016; Huang, Zhou, Li, Huang, & Wei, 2015; Moynihan, 1970; Poiani, 2010; Sommer & Vasey, 2006). Evidence of SSSB in *Colobus* is taken from a pers. comm. of Teichroeb in Pfau et al. (2019). The phylogeny was taken from the Open Tree of Life online resource (Hinchliff et al., 2015) and the divergence times from Pozzi et al. (2014). Stars indicate sequences that have been provided here in addition to those reported in Pfau et al. (Photo credits: Flickr and David Gonzales, Pexels; Christine Wehrmeier, Unsplash) (Color figure online)

The experimental evidence of *TRPC2* KO mice, combined with the loss of *TRPC2* in frequently SSSB-exhibiting cetaceans (Harvey, Dudzinski, & Kuczaj, 2017; Sommer & Vasey, 2006; Yu et al., 2010) and bats (Riccucci, 2011; Sugita, 2016; Yohe et al., 2017), provides a reasonable basis for supposing a homologous effect in Old World monkeys and apes (*Catarrhini*) as argued by Pfau et al. However, the evidence for such a connection is at present limited primarily by taxon sampling. Previous reconstructions establishing the ancestral pseudogenization of *TRPC2* only assessed up to 15 species as representatives of the respective 77 extant primate genera (Liman & Innan, 2003; Zhang & Webb, 2003). After mining GenBank to retrieve all possible sequences of *TRPC2*, we performed an updated reconstruction using 42 species of separate primate genera (Fig. 1 and supplementary information). We focused on a stop codon at position 71 in exon 13 of *TRPC2*, which was postulated by Pfau et al. to represent the ancestral loss of *TRPC2* function and hypothetical increase in SSSB in Old World monkeys. Using this larger sampling, and examining the distribution of this stop codon, we found that indeed the likely point of pseudogenization was after the split between the New World monkeys (*Platyrrhini*) versus Old World monkeys and apes (Fig. 1), as postulated by Pfau et al. We estimated this stop codon to have appeared between 46.7 and 32.1 million years ago (Mya; Fig. 1), slightly earlier than what was reported in Pfau et al. (i.e., 25 Mya). Other stop codons are found in exon 13 (supplementary information), although their distribution in fewer lineages would indicate that they appeared more recently than the premature stop at position 71. Ancestral state reconstructions for all stop codons indicated in the supplementary information were conducted using parsimony in Mesquite 3.6 (Maddison & Maddison, 2018). However, the pattern of SSSB and lost *TRPC2* function in primates does not map so easily.

SSSB has only been observed in three genera within *Strepsirrhini*, the lesser bushbabies (*Galago*), brown lemurs (*Eulemur*), and sifaka (*Propithecus*) (Bagemihl, 1999; Chandler, 1975), but in New World monkeys, at least 7 out of 19 genera are reported as exhibiting SSSB (Bagemihl, 1999; Carosi & Visalberghi, 2002; Dixson, 2012; Moynihan, 1970). Furthermore, the functionality of *TRPC2* in New World monkeys is still unclear. For example, spider monkeys (*Ateles*) appear to possess functioning *TRPC2* and yet also exhibit SSSB (Busia et al., 2018). Squirrel monkeys (*Saimiri*) and Atlantic forest marmosets (*Callithrix*) also perform SSSB, but they have an incomplete VNO with a reduced vomeronasal epithelium (VNE), through which *TRPC2* might not be able to express the phenotype for increased same-sex aversion (Pfau et al., 2019). Similarly, a reduced VNE has been reported in capuchins (*Cebus*), and interrupted or interspersed VNE in tamarins (*Saguinus*) and lion tamarins (*Leontopithecus)* (Smith et al., 2011), with all three species exhibiting SSSB (Bagemihl, 1999; Carosi & Visalberghi, 2002; Moynihan, 1970). Conversely, owl monkeys (*Aotus*) are reported to have a complex VNO (Pfau et al., 2019) and do not seem to exhibit SSSB (Hunter & Dixson, 1983), although the VNO of owl monkeys has also been described as small and unlikely to play a role in communication (Hunter, Fleming, & Dixson, 1984). Similarly, Smith et al. (2011) showed that SSSB-expressing lion tamarins possess a thicker VNE than owl monkeys (and described the owl monkey VNE as being poorly developed), thereby undermining the notion of VNE layers mediating *TRPC2* functionality and consequently SSSB. Both of the aforementioned VNO studies emphasize that the owl monkey VNO is similar in microanatomy to that of tamarins, an SSSB-exhibiting genus, and suggest that spider monkeys, which also exhibit SSSB, have the most similar VNO to the lemurs, which rarely, if ever, exhibit SSSB (Hunter et al., 1984; Smith et al., 2011). The pattern of SSSB expression in New World monkeys, therefore, cannot comfortably be coupled with variation in overall VNO structure.

## Absence of Evidence for Same-Sex Sexual Behavior is not Evidence of Absence

As mentioned above, SSSB is reported among New World monkeys in 7 out of 19 genera (Bagemihl, 1999; Carosi & Visalberghi, 2002; Dixson, 2012; Moynihan, 1970), but is believed to be substantially less frequent and less intense than in Old World monkeys and apes (Dixson, 2012). Although likely to be broadly true, caution should be taken when making such comparative statements, since studies are often non-equivalent, with different objectives, sampling effort, and variables; an analysis of mounting behavior alone might assess only a subset of mount frequency, latency, copulatory duration, intromission, and ejaculation. Functionality of *TRPC2* might better predict variation in frequency of SSSB, rather than presence–absence, but comparative studies of frequency and intensity of SSSB between primate genera are limited.

Generally, behavioral field studies of SSSB have only recently been substantially conducted, with early reports often taking the form of opportunistic anecdotes (Sommer & Vasey, 2006). For example, a recent study of spider monkeys reported the opportunistic observation of three homosexual couplings of one male with three different male partners as “low levels” of SSSB (Busia et al., 2018; Pfau et al., 2019), whereas the evidence for SSSB in wild Sumatran orangutans (*Pongo*) is comprised of opportunistic anecdotes involving mere two copulatory mounts (Fox, 2001). Furthermore, the relatively low frequencies of SSSB in primates other than Old World monkeys and apes do not explain the indisputably high frequencies observed in other mammals with functioning *TRPC2* and VNO, such as bison and red deer (Sommer & Vasey, 2006). We further argue that one cannot rely on behavioral studies unless they have been explicitly designed to assess SSSB, since without a mandate to observe SSSB, studies that report low frequencies or even absences may potentially be suffering from long-standing homophobic biases, or even the simple mistake of sexing individuals by presuming heterosexuality when any sexual coupling between individuals is observed (Bailey et al., 2016; Sommer & Vasey, 2006).

## Can a Premature Stop Codon in *TRPC2* be Compensated for?

*TPRC2* is considered non-functional because of a premature stop codon, but newly discovered mechanisms have shown that the function(s) of one gene with premature stop codons are frequently compensated for by the upregulation of orthologues from the same gene family (Peng, 2019). This discovery initially hinged on the fact that deleterious mutations with premature stop codons often only give a reduction in the relevant phenotype compared with the effects of acute knockdowns (reduced expression) of the same genes (Rossi et al., 2015). It now turns out that RNA transcripts with premature stop codons are preferentially degraded and gene family orthologues upregulated (El-Brolosy et al., 2019; Ma et al., 2019). This compensatory mechanism requires transcription of the mutant gene (RNA capping), and also the COMPASS complex, which catalyzes the methylation of histones at the transcriptional start site of upregulated gene orthologue family members. If there is no transcription of the mutant gene (for example, it is deleted entirely), there is no genetic compensation. Thus, a still unaddressed but critical issue is: could other *TRPC* gene orthologues be partially rescuing, perhaps tissue specifically, the phenotype of the *TRPC2* gene KOs with premature stop codons?

## Conclusion

To fully understand any behavior (here SSSB), it is important to distinguish between proximate hypotheses (“how it works”) from ultimate hypotheses (the “why” question). We note that Pfau et al. refer to the link between non-functional *TRPC2* and SSSB, and its loss in primate lineages, as an *ultimate* explanation; however, these are instead proximate hypotheses. Ultimate explanations require the expected fitness consequences of trait variation to be defined (Scott-Phillips, Dickins, & West, 2011). Since the proximate link between *TRPC2* and SSSB in primates remains unclear, we believe its clarification should be a priority for investigators in this field. This is not to say that the proposed explanation of group cohesion through socially adaptive functions facilitated by SSSB (which is an ultimate hypothesis) and/or pleiotropy with reduced aggression is unappealing, but we also note that a preliminary question, for example, would be to ask why *TRPC2* loss has not then been documented in the multi-male multi-female group-living diurnal lemurs, squirrel monkeys, and capuchins (Sussman, 1999).

Given recent progress on genome editing in primates (e.g., CRISPR; Zhou et al., 2019), researchers might consider the possibility of *TRPC2* KO in New World monkeys or lemurs, although by deleting the gene entirely and not by introducing premature stop codons. If Pfau et al.’s theory is true, then those KO mutants should exhibit increased SSSB. One might even attempt to rescue the function of *TRPC2* in an Old World monkey or ape and thereby expect suppression of SSSB, given that the true redundancy of their VNO has been contended (D’Aniello, Semin, Scandurra, & Pinelli, 2017). These investigations would need effective ethical oversight, not only because of animal welfare, but also because under no circumstances should *TRPC2* be advocated as a way to “cure” homosexuality. SSSB is likely under the control of multiple genes (Ratnu et al., 2017; Rice et al., 2016; Sanders et al., 2017), but linking *TRPC2*, VNO, and SSSB represents an exciting hypothesis by which genes can fine-tune the development of complex behaviors.

## Electronic supplementary material

Below is the link to the electronic supplementary material.

Supplementary material 1 (DOCX 305 kb)
