# Supplementary material for: The De-Scent of Sexuality: Should We Smell a Rat?
Source: Arch Sex Behav. 2019 Dec 5;50(6):2283–8. doi: 10.1007/s10508-019-01591-z (PMC8416816; doi:10.1007/s10508-019-01591-z)
Supplement: Supplementary file 1 — Supplementary material 1 (DOCX 305 kb) [file 10508_2019_1591_MOESM1_ESM.docx]

**SUPPLEMENTARY INFORMATION**

Alignment and in-frame stop codons in exon 13 in *TRPC2* for 42 primate genera, with reading frame established from functional *TRPC2* in mice and rats. *Pongo* and *Cercopithecus* share an earlier stop codon at position 40, but this appears to be the result of independent transitions. Genbank accession numbers are as follows: *Homo*/NR_002720; *Pan*/AH013039; *Gorilla*/AH013041; *Pongo*/AH013042; *Nomascus*/XM_012503222; *Hylobates*/AY302620; *Cercocebus*/XM_012045186; *Mandrillus*/XM_011993732; *Papio*/AH013038; *Theropithecus*/NC_037682; *Macaca*/XM_015436005; *Cercopithecus*/PVKI010037953; *Erythrocebus*/PVJV010042386; *Chlorocebus*/NC_023642; *Colobus*/AH013040; *Piliocolobus*/NW_020556228; *Rhinopithecus*/XM_010366924; *Pygathrix*/PVHW010015823; *Semnopithecus*/PVII010025336; *Saguinus*/AH013047; *Callithrix*/XM_003734180; *Aotus*/NW_018503629; *Saimiri*/AH013046; *Cebus*/XM_017532913; *Ateles*/AH013045; *Alouatta*/AY463534; *Pithecia*/AH013044; *Callicebus*/AY231374; *Plecturocebus*/PVKP010061601; *Carlito*/XM_008071159; *Cheirogaleus*/PVHR01012582; *Mirza*/PVHQ01017475; *Microcebus*/XM_020286595; *Propithecus*/XM_012645570; *Indri*/RJWJ010000934; *Eulemur*/PVJU010023829; *Prolemur*/MPIZ01000287; *Lemur*/PVHV01000848; *Varecia*/AY231370; *Daubentonia*/PVJZ01000091; *Nycticebus*/PVIV010016166; *Otolemur*/XM_023514071; *Mus*/NC_000073; *Rattus*/NC_005100.
